# [Expression of Concern] Insulin-like growth factor-1 receptor knockdown enhances radiosensitivity via the HIF-1α pathway and attenuates ATM/H2AX/53BP1 DNA repair activation in human lung squamous carcinoma cells

**DOI:** 10.3892/ol.2026.15543

**Published:** 2026-03-23

**Authors:** Xiaoxing Liu, Haiyan Chen, Xin Xu, Ming Ye, Hongbin Cao, Lei Xu, Yanli Hou, Jianmin Tang, Di Zhou, Yongrui Bai, Xiumei Ma

Oncol Lett 16: 1332–1340, 2018; DOI: 10.3892/ol.2018.8705

Following the publication of the above paper, it has been drawn to the Editor's attention by a concerned reader that, in Fig. 3, the blots shown for the IGF1R experiment in Fig. 3A and the β-actin experiment in Fig. 3B were remarkably similar. A subsequent analysis of the data in this paper performed by the Editorial Office revealed that the β-actin blots featured in Fig. 3A subsequently reappeared in a paper written by different authors at different research institutes in the journal *CNS Neuroscience & Therapeutics*.

The authors have been contacted by the Editorial Office to offer an explanation for these apparent anomalies in the presentation of the data in this paper, and we are awaiting their response. Owing to the fact that the Editorial Office has been made aware of potential issues surrounding the scientific integrity of this paper, we are issuing an Expression of Concern to notify readers of these potential problems while the Editorial Office continues to investigate this matter further.

